# Developmental conservation of microRNA gene localization at the nuclear periphery

**DOI:** 10.1371/journal.pone.0223759

**Published:** 2019-11-04

**Authors:** Eralda Salataj, Chrysoula Stathopoulou, Róbert A. Hafþórsson, Christoforos Nikolaou, Charalampos G. Spilianakis

**Affiliations:** 1 Institute of Molecular Biology and Biotechnology—Foundation for Research and Technology Hellas, Heraklion, Crete, Greece; 2 Department of Biology, University of Crete, Heraklion, Crete, Greece; 3 Department of Molecular Biology and Genetics, Democritus University of Thrace, Alexandroupolis, Greece; 4 Department of Biology, Lund University, Lund, Sweden; Hirosaki University Graduate School of Medicine, JAPAN

## Abstract

microRNAs are of vital importance for the regulation of the adaptive and innate immune responses, modulating gene expression at the post transcriptional level. Although there is cumulative information regarding the steady state mature microRNA levels and their respective targets, little is known about the effect of the three-dimensional chromatin architecture on the transcriptional regulation of microRNA gene loci. Here, we sought to investigate the effect of subnuclear localization on the transcriptional activation of eight murine microRNA loci in the immune system. Our results show that microRNA genes display a preferential monoallelic gene expression profile accompanied with perinuclear localization irrespectively of their transcription status or differentiation state. The expression profile and perinuclear localization are developmentally conserved while microRNA gene loci localization outside constitutive lamin associated domains is cross-species conserved. Our findings provide support for an active nuclear periphery and its role in chromatin organization of the non-coding genome.

## Introduction

The last few years it has become increasingly clear that higher order chromatin organization controls the regulation of genome activity and serves as an additional epigenetic mechanism that modulates cellular functions and gene expression programs in diverse biological processes. Spatial positioning of different gene loci can be directly linked to gene expression [1–3] while other findings confirm that deregulation of the nuclear architecture can be linked to severe diseases [4–6]. Apart from the organization of chromatin *per se*, the metazoan interphase nuclei are also functionally compartmentalized, with different repressive and active nuclear sub-compartments governing gene expression. Compartments such as the nuclear lamina and the nucleolus gather repressed genes, while RNA Pol II factories attract expressing genes [7, 8]. Allelic interactions and gene repositioning with functional importance are common during the regulation of immune responses [9–11]. Chromatin loops, constituting Lamina Associated Domains (LADs), modify their proximity to the nuclear lamina offering a plausible explanation to past reports documenting the relocalization of gene loci, upon their transcriptional activation, away from the nuclear periphery, further supporting the implication of nuclear lamina in gene silencing [12].

In contrast to the nuclear lamina, association of chromatin with the Nuclear Pore Complex (NPC) has been reported to favor gene expression. Nuclear pore is a multiprotein complex of nucleoporins, that mediates molecule transport between the cytoplasm and the nucleus. Additional to molecule transportation, the NPC exerts multiple roles that are associated with regulation of gene expression, chromatin organization and virus infection [13–17].

Prior studies have introduced the role of non-coding RNAs as prominent factors that control gene expression and chromatin organization [18]. Non-coding RNAs can be subdivided into two discrete classes based on their physical properties (long and small non-coding RNAs). They are implicated in the regulation of several developmental and physiological processes in plants, fungi, worms and animals where they regulate gene expression, either through transcriptional modulation or via post-transcriptional regulatory mechanisms [19]. Although a plethora of reports have highlighted the significance of the long non-coding genome in nuclear organization [20], there are also few well characterized groups of small non coding RNAs with functional roles that can control genome stability and chromatin organization [21].

microRNAs constitute another abundant class of endogenous and highly conserved small non coding RNA molecules that show tremendous flexibility in controlling gene expression at the transcriptional and post-transcriptional level [22]. Several reports have described the functional role of microRNAs in controlling the myeloid and lymphoid cell development and function of the innate and adaptive immune system [23].

Mammalian microRNA gene transcription is initiated in the cell nucleus by either RNA Polymerase II (Pol II) or RNA Polymerase III (Pol III) and it may yield a several kb (pri-miRNA) transcript that is co-transcriptionally processed to an ~70 bp premature RNA (pre-miRNA) by the Microprocessor complex [comprised by DROSHA and DiGeorge syndrome critical region gene 8 (DGCR8) proteins] [24–28]. DICER further processes the pre-microRNA and releases a 20–24 nt RNA duplex ready to obtain its functional targeting role in the cytoplasm [29–31]. Despite the fact that microRNA biogenesis and the microRNA-mediated gene regulation mechanisms are well documented, little is known about the transcription regulation of the microRNA genes themselves. So far, only a few studies have referred to the subnuclear positioning of microRNA genes as an epigenetic mechanism regulating immune responses [32, 33].

Therefore, in this study we sought to investigate the impact of nuclear architecture as an epigenetic mechanism regulating the expression of eight microRNA genes in the murine innate and adaptive immune system. We investigated the role of subnuclear localization of microRNA gene loci as a potential mechanism affecting non-coding genome expression. Apart from highlighting the impact of the nuclear periphery in microRNA gene expression, we also introduce other factors that may modulate the subnuclear positioning or the expression of the microRNA genes studied. Our results pinpoint the impact of nuclear periphery in microRNA gene expression, shed light on chromatin organization at the nuclear periphery and allow for a better understanding of the mammalian genome organization.

## Results

### microRNA genes are preferentially expressed in a monoallelic manner

To study the impact of nuclear architecture in the transcriptional regulation of microRNA gene loci, eight microRNA genes (*miR-181a1b1*, *miR-181a2b2*, *miR-181c*, *miR-142*, *miR-146a*, *miR-17-92*, *miR-155* and *miR-let7e*) were selected which are expressed during the murine myeloid and lymphoid cell development. Initially, our study focused on unraveling the allelic expression profile of these microRNA genes. RNA-DNA fluorescence *in situ* hybridization (FISH) experiments were performed in T cells [murine thymocytes, CD4^+^, T helper type 1 (TH1) and type 2 (TH2) cells] and in macrophages (thioglycollate elicited peritoneal macrophages–TEPMs, and bone marrow derived macrophages–BMDMs) before and after lipopolysaccharide (LPS) stimulation.

**Fig 1:**
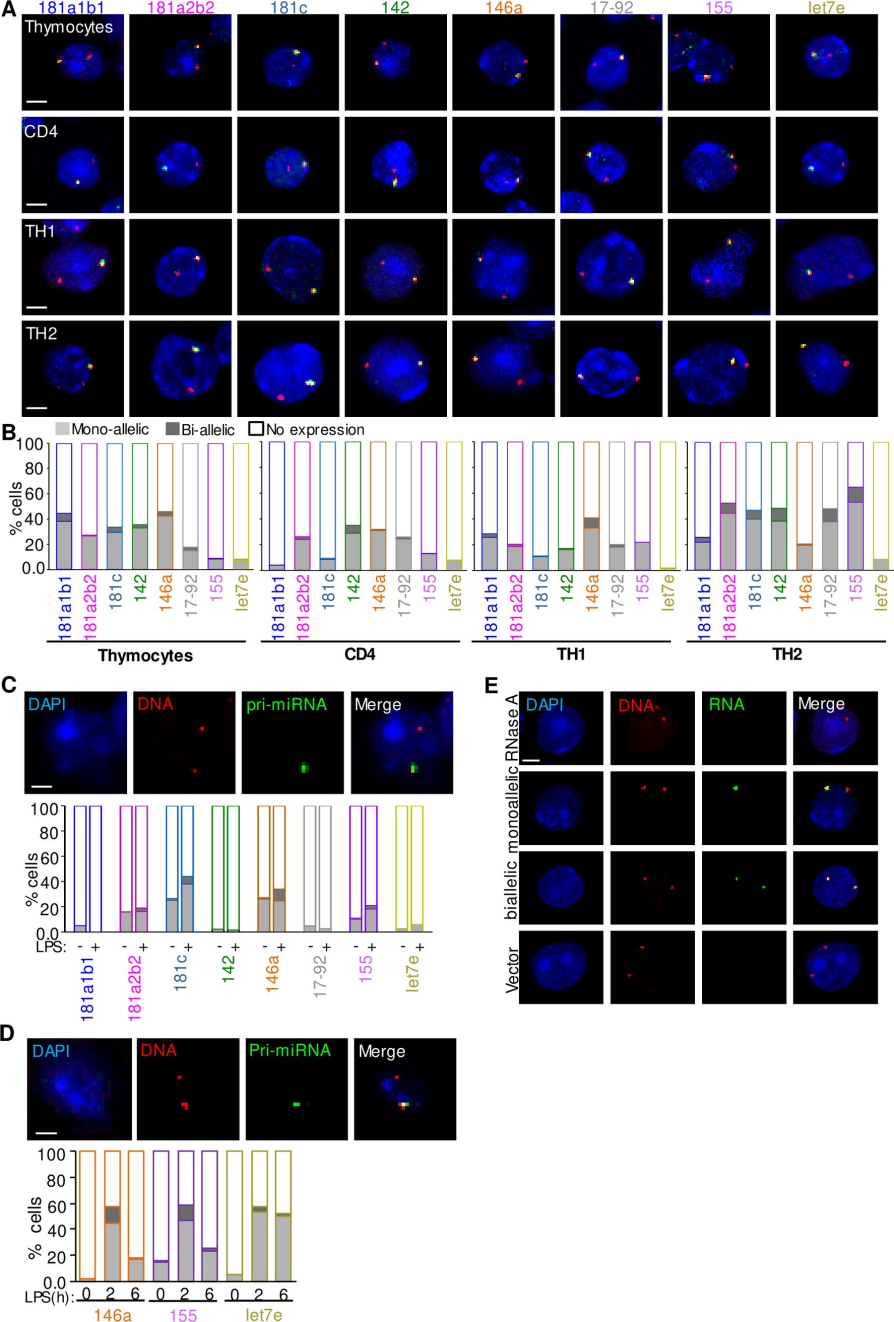
Allelic expression profile analysis of microRNA gene loci reveals their preferential monoallelic expression in the murine immune system. A) Representative images of RNA-DNA FISH experiments for microRNA genes in thymocytes, CD4^+^, TH1, TH2 cells. microRNA gene locus DNA (red) and biotinylated cDNA probes (green) were used to detect the nascent microRNA transcript. The number of nuclei measured in the RNA-DNA FISH experiments were: n = 1950 thymocytes, n = 2348 CD4^+^, n = 3417 TH1, n = 3057 TH2. Scale bar 2μm. B) The mono- and bi-allelic expression profile of the portrayed microRNAs in the graph corresponds to the percentage of cells expressing either one or both alleles, before and after differentiation of the cells into the TH1 and TH2 cell lineages. The number of nuclei measured are the same as in Fig 1A. C) Representative confocal microscopy images of RNA-DNA FISH experiments showing the allelic expression profile of microRNA genes in thioglycollate elicited peritoneal macrophages (TEPMs). Bar graphs indicate the percentage of cells with either mono- or bi-allelic expression of the nascent pri-miRNA transcript before and after LPS stimulation of macrophages. A total number of 3558 and 2606 nuclei were measured in naive (-LPS) and LPS activated (+LPS) TEPMs respectively. Scale bar 2μm. D) Confocal microscopy single z-stack images indicating the colocalization of nascent *pri-miR-155* transcript (green) along with its corresponding gene locus (red), in 2h LPS-stimulated BMDMs (blue-DAPI, for DNA counterstain). Bar graph portraying the mono- and bi-allelic expression pattern of *miR-146a*, *miR-155* and *miR-let7e* in naive and LPS-stimulated BMDMs. The total number of nuclei measured for each microRNA locus were n = 691 for *miR-146a*, n = 931 for *miR-155* and n = 168 for *miR-let7e*. Scale bar 2 μm. E) Representative confocal microscopy images of RNA-DNA FISH experiments for microRNA gene loci (DNA- *miR-155* red) with the respective nascent pri-microRNA transcript (green) in thymocytes (DNA counterstained with DAPI—blue). The upper panel depicts DNA, but not RNA signals in RNase A treated cells. The second and third panel depict mono- and biallelically expressing cells. The fourth panel presents RNA-DNA hybridization with the TOPO-TA cloning vector used for RNA FISH probe synthesis. Scale bar 2 μm.

Our results revealed a profound tendency for monoallelic microRNA gene expression both in T cells (Fig 1A and 1B) and macrophages (Fig 1C and 1D). RNA-DNA FISH experiments allowed the simultaneous detection of both the newly synthesized pri-miRNA and the gene locus from which it is transcribed (Fig 1E). Upon terminal differentiation of CD4^+^ T cells into the TH1 and TH2 cell lineages and after LPS stimulation of TEPMs or BMDMs, the expression of microRNA genes remained monoallelic although a low fraction of cells exhibited biallelic expression. The higher frequency of monoallelically expressed microRNA gene alleles is due to the low expression levels of primary microRNA transcripts and not due to imprinting. These results are in line with bioinformatic predictions regarding the allelic expression profile of both microRNA genes and coding genes and does not seem to be affected by neighboring gene transcription status (S1 Fig and S1 Table).

### microRNA gene loci are perinuclearly positioned and colocalize with the nuclear lamina

**Fig 2:**
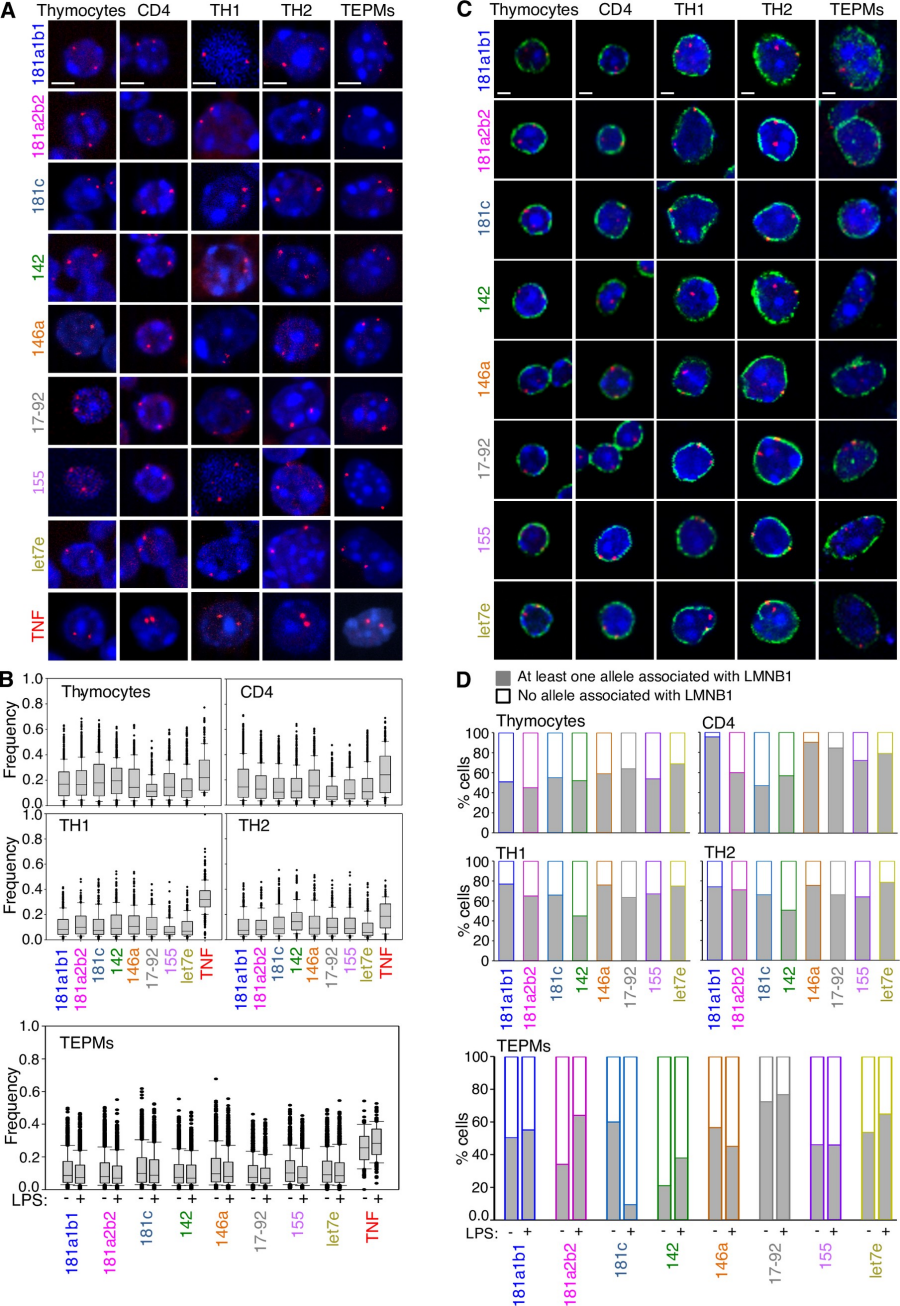
microRNA gene loci are localized in the cell nuclear periphery. (A) DNA FISH single z-stack images displaying the perinuclear localization of microRNA and *Tnfα* gene loci in thymocytes, CD4^+^, TH1, TH2 and TEPMs. Scale bar 2μm. (B) Bar graphs represent the intranuclear normalized distances (NDs) measured between the microRNA gene signals and the edges of the nuclei as defined by DAPI staining. Allele distances from the nucleus edge were measured in 3D-preserved cell nuclei using the Volocity 3D Image Analysis Software and normalized to the nuclear radius. ND = 1 defines the center of the nucleus, whereas ND = 0 defines the nuclear periphery. The total number of nuclei measured in each dataset were: Thymocytes n = 4021, CD4^+^ n = 4030, TH1 n = 1337, TH2 n = 1331, TEPMs/-LPS n = 4762, TEPMs/+LPS n = 4882. Scale bar 2μm. (C) Single z-stack images of DNA FISH combined with immunofluorescence analysis indicating the colocalization of microRNA gene alleles (red) with the nuclear lamina (LMNB1 green) in thymocytes, CD4^+^, TH1, TH2 cells and TEPMs. Scale bar 2μm. (D) Bar graphs represent the percentages of cells bearing at least one allele associated with the nuclear lamina. Number of nuclei assessed: thymocytes n = 2008, CD4^+^ n = 1984, TH1 n = 1232, TH2 n = 1320, TEPMs/-LPS n = 958, TEPMs/+LPS n = 1151.

Collectively, the RNA/DNA FISH analysis on three-dimensionally (3D) preserved cell nuclei, provided us with interesting preliminary data indicating that microRNA gene loci localize in the nuclear periphery. Considering that the subnuclear localization of gene loci can directly impact their gene expression [11, 34] we performed 3D-DNA FISH experiments in murine T and macrophage cell lineages (Fig 2A). The *Tnfα* gene locus was used as a control, given that its expression displays well characterized spatiotemporal kinetics [11]. Intranuclear distances (between each gene allele and the nuclear periphery) were measured and normalized to the nuclear radius of each cell in order to correct for differences in the size of the cell nuclei. To quantitate the relative localization of microRNA gene loci in cell nuclei, the normalized distances (NDs) of gene alleles were grouped in ten concentric shells, in which normalized distances ranging from 0 to 1 are denoting the border and the center of the cell nucleus respectively (S2A Fig). Thymocytes and CD4^+^ T cells demonstrated a peripheral distribution for all eight microRNA gene loci, with the most peripheral cluster ND = 0–0.1 including more than 40% of all the microRNA gene alleles (Figs 2B and S2B and S1 Video). Upon differentiation of CD4 cells into TH1 and TH2 cells the external cluster ND = 0–0.1 included as much as 65% and 60% of the total microRNA gene alleles, respectively. Although microRNA gene loci displayed a perinuclear relocalization during differentiation, *Tnfα* alleles exhibited an expanded distribution up to a more internal cluster ND = 0.6–0.7 with a low percentage of the alleles (15%) located at the nuclear periphery (ND = 0–0.1) in thymocytes. During T cell differentiation, *Tnfα* was relocalized towards the center of the nucleus in TH1 cells (ND = 0.3–0.4 including the highest fraction of alleles) while in TH2 cells the allelic distribution of *Tnfα* was similar to CD4^+^ cells.

A perinuclear localization of microRNA gene alleles was also observed in TEPMs (Figs 2B and S2B) and BMDMs (S2C Fig). More specifically, in TEPMs irrespectively of LPS stimulation, more than 50% of the microRNA gene alleles localized in the nuclear periphery (0–0.1). As for the *Tnfα* alleles localization, the corresponding *Tnfα* allelic ND values clustering in the ND = 0–0.1 zone were 5.8% and 1.5% before and after LPS stimulation respectively, following a reverse distribution pattern compared to the microRNA genes. The same perinuclear positioning of the microRNA genes was observed in BMDMs (S2C Fig). These results indicate that in cell types of the murine innate (macrophages) and adaptive (CD4^+^ T cells) immune system, the eight microRNA gene loci under study preferentially localize in the nuclear periphery irrespectively of the differentiation or activation state of the cell.

In order to experimentally test whether the peripheral distribution of the investigated microRNA gene loci was attributed to a dynamic interplay with the nuclear lamina, as already described for coding genes [35], we performed 3D-DNA FISH in concert with immunofluorescence experiments and assessed the fraction of cells with at least one gene allele colocalized with the nuclear lamina (Fig 2C). We found that more than 50% of the thymocytes displayed at least one microRNA gene allele colocalized with Lamin-B1 (Fig 2D and S2 Video). Upon activation and differentiation of CD4^+^ T cells towards the TH1 and TH2 cell lineages, the fraction of alleles that colocalized with Lamin-B1 was increased with over 50% of the cells displaying at least one microRNA gene allele colocalized with Lamin-B1 (Fig 2D). Our experiments in TEPMs indicated that for *miR-181a1b1*, *miR-181c* (unstimulated cells), *miR-17-92* and *miR-let7e*, more than 50% of the total cell population bore at least one allele colocalized with the nuclear lamina. *miR-181a2b2*, *miR-146a* and *miR-155* colocalized with the nuclear lamina in cell fractions higher than 40% while *miR-142* exhibited a lower tendency to colocalize with Lamin-A/C (20%). In BMDMs we tested the microRNA genes for which the expression was upregulated upon LPS stimulation and found that at least one allele colocalized with Lamin-B1 in more than 50% of the total cell population assessed (S2D Fig). In conclusion, the observed colocalization of microRNA gene loci with the nuclear lamina did not significantly change before and after T cell differentiation or macrophage LPS stimulation with the striking exception of the LPS-induced *miR-181c* gene locus in TEPMs (Fig 2D), which demonstrated the lowest percentage of colocalization with the nuclear lamina in CD4^+^ cells and LPS stimulated TEPMs.

### microRNA genes are actively transcribed in the nuclear lamina

**Fig 3:**
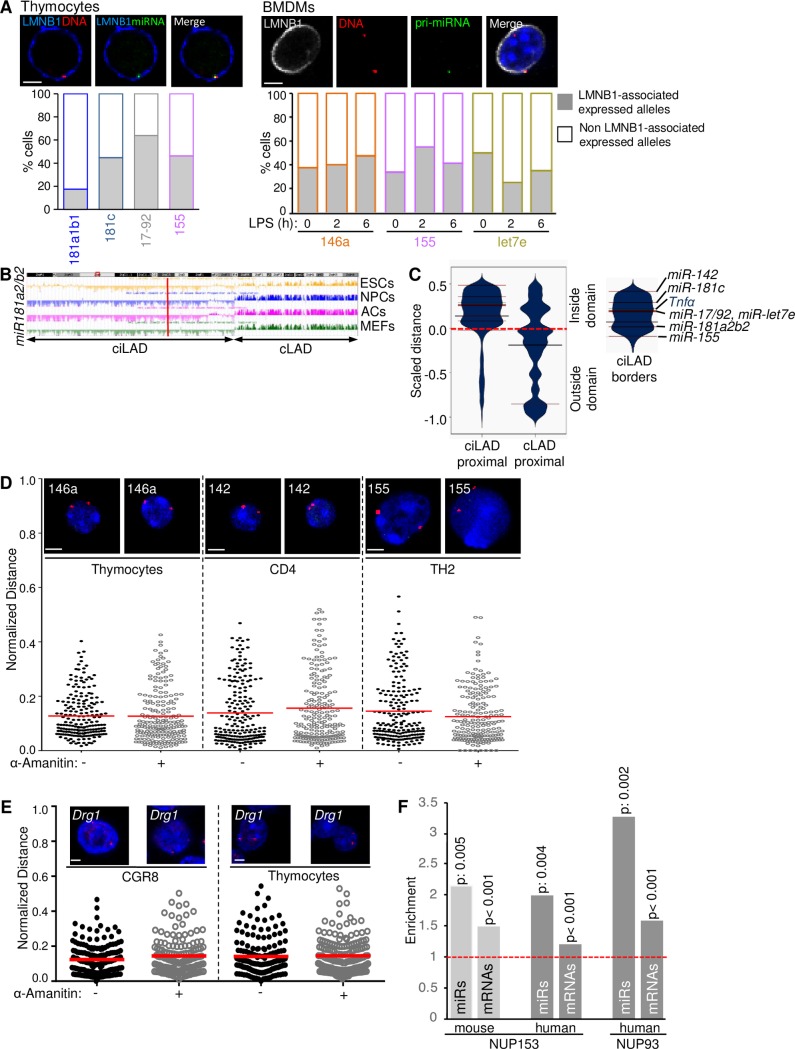
The perinuclear localization of microRNA gene loci is independent of their transcriptional activity. (A) Single z-stack images displaying the colocalization of expressed (green) microRNA gene alleles (red) with Lamin-B1 in thymocytes and BMDMs (before and upon LPS stimulation for the indicated time points). Bar graphs represent the percentage of expressed alleles colocalized with the nuclear lamina. Number of nuclei assessed: thymocytes n = 705, BMDMs/-LPS n = 492, BMDMs/+2h LPS n = 1128, BMDMs/+6h LPS n = 1028. Scale bar 2μm. B) 2Mbp region of mouse chromosome 2 with *miR-181a2b2* gene. ciLAD/cLAD analysis in four distinct cell types, embryonic stem cells (ESCs), neural progenitors (NPCs), astrocytes (ACs) and mouse embryonic fibroblasts (MEFs). (C) Beanplots portraying the scaled distance distribution of microRNA genomic sequences to their most proximal ciLAD/cLAD regions. Red lines represent the microRNA genes under study, whereas the green line corresponds to TNF*α* locus. Small but interesting tendencies for the localization of microRNA sequences either towards the centers of ciLADs, or at the cLAD/ciLAD boundaries are observed and recapitulated by the Wilcoxon rank-sum test (p-value < = 10e-90). (D) Single z-stack images of 3D DNA-FISH nuclei and scatter plot indicating the ND distribution of microRNA gene alleles before and upon transcriptional inhibition with *α*-amanitin treatment in thymocytes, CD4^+^ and TH2 cells. Number of nuclei assessed: thymocytes n = 400, CD4^+^ n = 400, TH2 n = 398. Scale bar 2μm. (E) Scatter plot indicating the ND distribution of the cLAD localized *Drg1* gene before and after *α*-amanitin treatment of CGR8 mouse embryonic stem cells and thymocytes. Number of nuclei assessed: CGR8/(-)*α*-amanitin n = 170, CGR8/(+)*α*-amanitin n = 144, thymocytes/(-)*α*-amanitin n = 158, thymocytes/(+)*α*-amanitin n = 164. (F) Assessment of overlap tendencies between a) microRNA and b) protein coding mRNA gene loci with peaks corresponding to human and mouse nucleoporins. Enrichment of colocalization was calculated as observed over expected ratios of overlaps between the compared genomic regions. P-values were calculated on the basis of 1000 random permutations of the peaks’ regions. Values <0.001 indicate not one out of 1000 permutations had a value as high as the observed.

In order to investigate the repercussions of microRNA gene colocalization with the nuclear lamina and its effect on the allelic microRNA gene expression profile, we performed IF-RNA/DNA FISH (for the pri-microRNA and the gene locus) and assessed the frequency of microRNA gene alleles that were actively expressed and were either colocalized or not with the nuclear lamina (either LMNB1 or LMNA/C). Our results indicated expression and collateral colocalization with Lamin-B1 for all microRNA genes tested. More specifically, in thymocytes, almost 45% of the expressing alleles of *mir-181c*, *miR-17-92* and *miR-155* colocalized with Lamin-B1 while in macrophages before and after LPS stimulation more than 40% of all the *miR-146a* and *miR-155* expressing alleles were colocalized with Lamin-B1 (Fig 3A). The latter was also confirmed by the distributions of normalized distances between the expressed and the non-expressed microRNA gene alleles in thymocytes, CD4^+^, TH1, TH2 (S3A Fig), TEPMs and BMDMs before and after LPS stimulation (S3B Fig). We did not detect any tendency for localization of the expressing microRNA gene alleles to more internal nuclear compartments or *vice versa*.

To obtain a global view on the microRNA perinuclear positioning we tested whether the peripheral localization could be attributed to the localization in lamin associated domains (LADs). LADs are AT-rich, genomic regions known to constitute anchorage domains of gene loci to the nuclear lamina compartment and constitute almost 40% of the mouse genome [12, 36, 37]. Data obtained from DamID experiments on different cell types showed three different groups of LADs: constitutive LADs (cLADs) as regions that are lamin-associated regions, constitutive inter-LADs (ciLADs) that are chromatin regions between LADs and facultative LADs (fLADs) that are cell-type dependent lamin-associated regions [36]. Using published cLAD coordinates in the mouse genome [12], we tracked the position of the eight microRNA gene loci under study along with the TNF*α* locus in four different murine cell types: mouse embryonic stem cells (ESCs), mouse embryonic fibroblasts (MEFs), neuronal progenitor cells (NPCs) and astrocytes (ACs). We observed that all gene loci tested, resided outside cLAD regions in the mouse genome (Fig 3B). Subsequently, we questioned whether the observed perinuclear localization of microRNA gene loci reflected their localization in proximity to the borders of ciLADs. Our analysis revealed that *miR-181a2b2*, *miR-17-92*, *miR-155* and *miR-let7e* were localized closer to the ciLAD boundary, whereas *miR-181c*, *miR-142* and *Tnfα* gene loci were localized towards the center of their corresponding domains (Fig 3C). When the comparative analysis was scaled up to all the murine microRNA genes annotated in the miRbase, we found that microRNA gene loci did not exhibit a distinct tendency of localization towards either the border or the center of ciLAD/cLAD compartments (Fig 3C). microRNA genes located proximal to cLADs were positioned far from the corresponding boundaries, whereas ciLAD-associated microRNA genes were preferentially positioned within the domain, exhibiting a slight preference towards the center of the domain.

It is well established [38–40] that upon transcriptional activation genes may re/localize within the cell nucleus. Furthermore, during development and differentiation of T cells, a dynamic change in chromatin is observed [41–44]. Based on these findings, we next tested whether transcription inhibition could have an impact on the perinuclear localization of a microRNA gene locus or whether it would induce its relocalization. Therefore, we performed DNA FISH experiments in T cells, before and upon treatment with *α*-amanitin for 6h (Fig 3D). The efficiency of transcription inhibition was confirmed by both qRT-PCR and RNA FISH for all the cell types under investigation (S3C and S3D Fig). Our results demonstrated that in thymocytes, inhibition of transcription did not alter the gene allelic distribution of *miR-146a* locus. 48% of the gene alleles located at the nuclear periphery (ND = 0–0.1) in untreated cells compared to 50% of the gene alleles in *α* -amanitin treated cells. For *miR-142*, 46% of the alleles in untreated CD4 cells were located at the nuclear periphery (ND = 0–0.1) compared to 51% of the gene alleles being perinuclear in *α*-amanitin treated cells. In TH2 cells 48% of *miR-155* gene alleles were perinuclear (ND = 0–0.1) in untreated compared to 50% in treated cells (Fig 3D). Furthermore, Kolmogorov Smirnov (KS) analysis of the normalized distances for each population displayed a non-significant difference before and after *α*-amanitin treatment of cells (S3E Fig). We also performed the same experiments for a cLAD-located gene [Developmentally-regulated GTP-binding protein 1 (*Drg1*)] in both ES cells and thymocytes. Our results indicated that trancription inhibition did not cause any statistically significant relocalization of *DRG1* alleles towards the nuclear interior in either of the two cell types (Figs 3E and S3F). In conclusion, the *α*-amanitin-dependent inhibition of active transcription did not affect the perinuclear localization of either the ciLAD-located microRNA genes, nor the cLAD-located control coding gene (*Drg1)*.

Based on the previous results, we concluded that the microRNA gene loci under study localize in the nuclear periphery in close proximity to the nuclear lamina, irrespectively of their transcription status. Therefore, we questioned whether microRNA genes colocalize with NPCs. Such an interaction of NPCs with euchromatin could promote transcription and control chromatin organization as previously reported [45–47]. We analyzed publicly available ChIP-seq data for NUP153, NUP93 and NUP98 (GEO numbers are provided in the corresponding Supporting information section). We found that microRNA gene loci were significantly enriched, compared to coding genes, for NUP153 and NUP93 proteins (Fig 3F). While NUP153 and NUP93 displayed similar results, this was not the case for NUP98 where we did not observe any significant enrichment between microRNA gene loci and mRNAs (S3G Fig). Based on these results we conclude that nuclear pore proteins may be implicated in tethering microRNA gene loci to the nuclear periphery.

### Ablation of the BACH1 transcriptional regulator does not affect the expression or peripheral localization of microRNA genes

**Fig 4:**
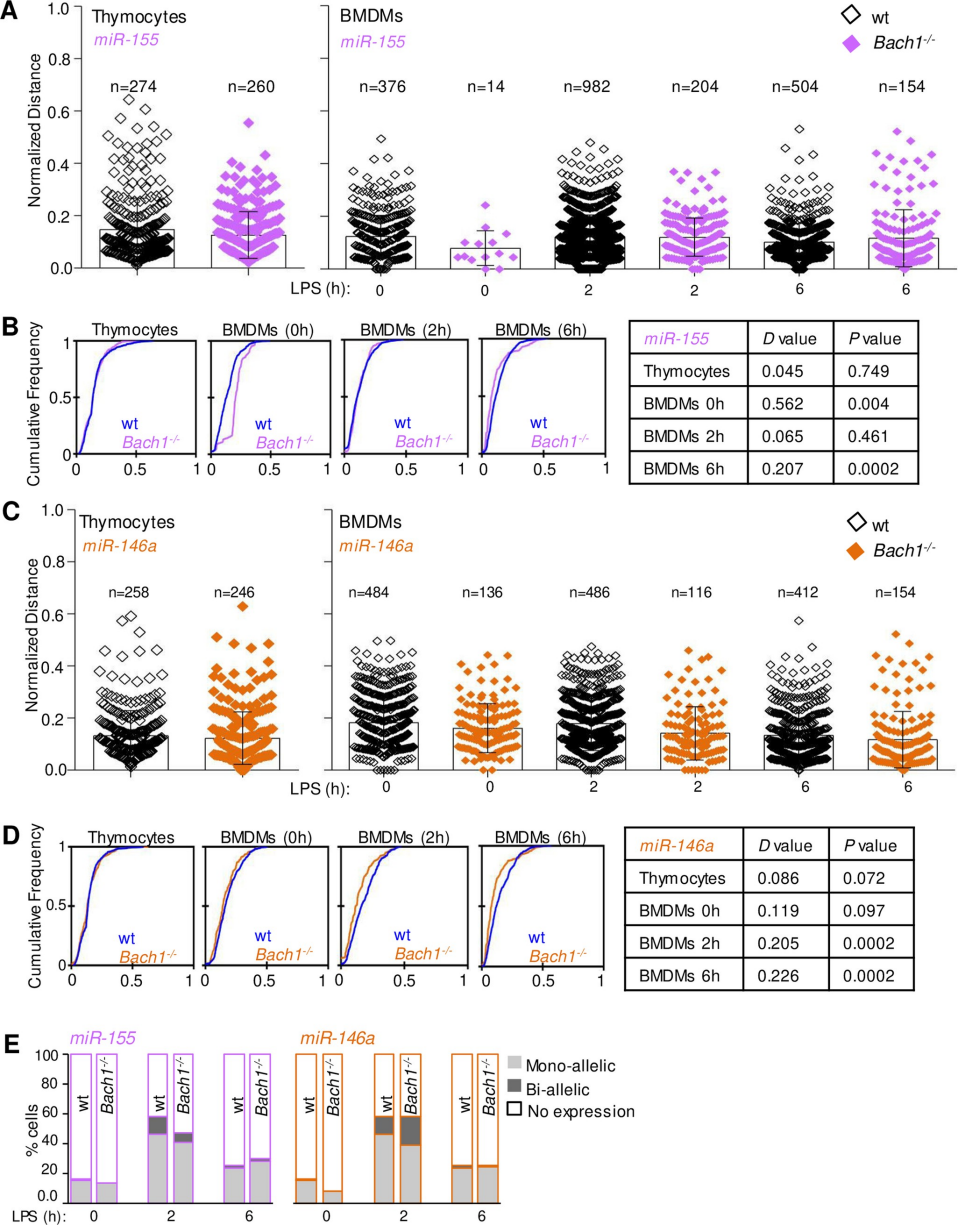
Perinuclear localization of microRNA gene loci in *Bach1*^*-/-*^ thymocytes and BMDMs. (A) *miR-155* gene alleles distribution in wild type (wt) and *Bach1*^*-/-*^ thymocytes and BMDMs. (B) Cumulative frequency graphs and KS-test for the comparison of *miR-155* gene alleles distribution in wt versus *Bach1*^*-/-*^ thymocytes and BMDMs before and after LPS stimulation for the indicated time points. (C) *miR-146α* gene alleles distribution in wt and *Bach1*^*-/-*^ thymocytes and BMDMs. (D) Cumulative frequency graphs of *miR-146α* gene alleles NDs in wt and *Bach1*^*-/-*^ thymocytes and BMDMs before and after LPS stimulation for the indicated time points. KS-test analysis results are portrayed on the table. (E) Allelic expression profile of *pri-miRNA-155 and pri-miRNA-146α* in naive (0h) and LPS stimulated (2h, 6h) BMDMs, following RNA-DNA FISH.

Cell type specific transcription factors may be implicated in gene positioning and spatial chromatin organization [34, 48–50]. BACH1 is a transcription factor highly expressed in the bone marrow and was characterized as an architectural transcription factor mediating chromatin interactions among Maf recognition elements-MAREs [51]. Additionally, *Bach1* deletion has a significant impact on lymphoid- and myeloid-mediated inflammatory responses [52] and its binding is enriched on microRNA gene promoters [53]. To investigate whether the ablation of BACH1 could affect either the subnuclear localization, or the allelic expression profile of microRNA genes, we performed DNA FISH experiments in both wild type (wt) and *Bach1*^*-/-*^ thymocytes and BMDMs and analyzed the subnuclear distribution of *miR-155* gene alleles, which we previously found to localize in the nuclear periphery. Our analysis in *Bach1*^*-/-*^ reconfirmed the peripheral localization of *miR-155* and *miR-146a* gene alleles in thymocytes and BMDMs that was not statistically different between wild type (wt) and *Bach1*^*-/-*^ cells (Fig 4A–4D).

To test whether *Bach1* ablation impacted microRNA transcriptional activation after LPS stimulation, we analyzed the allelic expression profile of *miR-155* and *miR-146a* in wild type and *Bach1*^*-/-*^ BMDMs with *in situ* pri-microRNA hybridization (Fig 4E). Our analysis revealed that *Bach1* ablation in BMDMs did not affect the allelic expression profile or subnuclear localization of *miR-155* and *miR-146a* genes.

### Developmentally conserved perinuclear localization of microRNA gene loci

**Fig 5:**
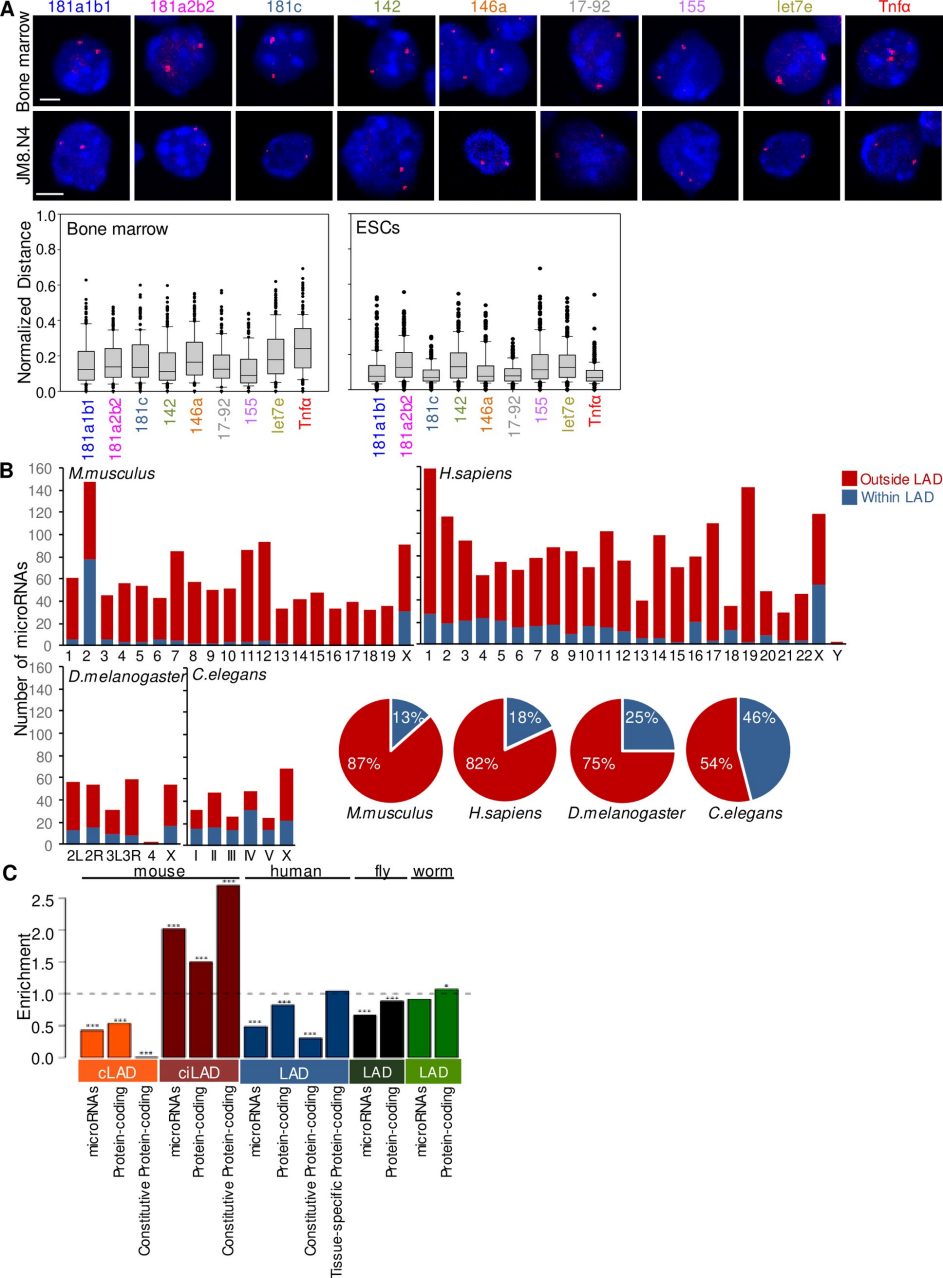
The perinuclear localization of microRNA gene loci is developmentally conserved. (A) Representative 3D DNA FISH confocal images in bone marrow and JM8.N4 ESCs. Number of nuclei assessed: bone marrow n = 200 alleles, ESCs n = 260 alleles. Scale bar 2μm. (B) Number of microRNA genes located within and outside cLADs per chromosome (Bar graphs) and total percentage of cLAD/ciLAD microRNA gene colocalization (Pie charts) in *M*.*musculus*, *H*. *sapiens*, *D*.*melanogaster* and *C*.*elegans*. (C) Enrichment values of microRNA and protein-coding genes within and outside LAD regions. Permutation *p*-values are depicted as ***:<0.001, **:<0.01 and *:<0.05 respectively, and calculated on the basis of 1000 permutations.

Based on the consistency of our findings regarding the perinuclear positioning of microRNA genes tested, we next investigated whether this preferential localization constitutes an immune cell specific phenomenon or has been established in earlier developmental stages in a manner that excludes the tested microRNA genes from internal nuclear positions. We examined the allelic distribution of the aforementioned microRNA gene loci in whole bone marrow derived cells (composed of myeloid precursors, eosinophils, basophils and monocytes, erythroid progenitors and lymphocytes) and in mouse embryonic germline competent cells (JM8.N4 and CGR8 cell lines). The allelic distribution of microRNA gene loci compared to the *Tnfα* gene locus in bone marrow cells displayed a similar pattern with T cells and macrophages. Gene allele frequencies in the 0–0.1 ND cluster ranged between 28% (*miR-146a*) to 57% (*miR-155*), while *Tnfα* alleles were mainly distributed (*56%*) in the 0.2–0.4 ND cluster. In ESCs microRNA gene alleles were mostly located to the nuclear periphery, with more than 80% of the alleles occupying the most perinuclear cluster (ND = 0–0.1) (Figs 5A and S4A–S4C). Interestingly, the *Tnfα* locus also demonstrated a perinuclear localization in ESCs with 80% of its gene alleles ND frequencies included in the most perinuclear cluster (ND = 0–0.1). In conclusion, although the *Tnfα* gene locus gradually relocalized from the nuclear periphery (ESCs) to the nuclear interior during myeloid and lymphoid differentiation (T cells, TEPMs, BMDMs), microRNA gene loci constantly displayed a perinuclear localization in cell lineages ranging from embryonic stem cells to more differentiated cell types of the innate and adaptive immune system.

It was previously suggested that cLAD domains are highly conserved across species, while ciLAD domains show a strong conservation between mouse and human [36]. Based on these reports we next investigated the correlation between microRNA gene localization and cLADs in four different species (*M*.*musculus*, *H*.*sapiens*, *D*.*melanogaster* and *C*.*elegans)*, details on the LAD coordinates are provided in the corresponding Supporting information section) [12, 54–56]. Our results indicated that in *M*.*musculus* 14% of the microRNA genes were localized within cLAD regions while the rest were positioned within ciLADs. Interestingly, chromosome 2 which contains a cluster of microRNAs that spans an area of around 50000bp, displayed an unusual high number of microRNAs inside cLADs, with 78 microRNA genes positioned within cLADs from a total of 147 microRNA genes (Fig 5B). *In silico* analysis revealed that, similarly to *M*.*musculus*, *H*.*sapiens* microRNA genes displayed a tendency to avoid the constitutive LAD domains. We found that 18% of microRNA genes were located within cLADs while the rest were located within ciLADs. Chromosome X displayed the highest number of microRNA genes localized within a cLAD region (54 out of 118 microRNAs). Chromosome 19, displaying the highest gene density, demonstrated the highest number of microRNA genes outside cLADs (Figs 5B and S4D). *D*.*melanogaster* demonstrated a similar pattern as observed in humans and mice, with only 25% of microRNAs located within cLAD regions. *C*. *elegans* was the only species with a different distribution of cLAD/ciLAD-containing microRNA genes, where 47% of the microRNA genes were located within cLADs (Fig 5B).

We expanded our comparative analysis and investigated the relative enrichment of all microRNA and protein coding genes in LADs. We next tested whether the observed enrichment of microRNA genes outside cLADs exhibited significant cross-species conservation. Our results in the mouse indicated that both microRNA and protein coding gene loci were significantly enriched in ciLAD regions (Fig 5C). We found significant depletion of microRNA gene loci in LADs except for the *C*.*elegans* dataset where the observed depletion of microRNA gene loci in LADs was not significant. The same trend was identified for protein-coding genes, albeit to a lesser extent among the four compared species, with enrichment values being below the baseline (value 1), but always higher than the corresponding ones for microRNA genes. This result is indicative of a cross-species conserved increased tendency of microRNA gene loci to avoid LAD regions.

## Discussion

Our study provides substantial information on the allelic expression profile of eight microRNA genes implicated in the regulation of the murine innate and adaptive immune responses. We have attributed the monoallelic expression pattern to the moderate expression levels of these genes [57, 58] and excluded the potential of imprinting for the aforementioned loci, since low frequency biallelic expression was detected in cell populations with maximal pri-microRNA expression, following T cell receptor (TCR) activation of CD4^+^ T cells and LPS stimulation of macrophages [59]. Although monoallelic expression has been reported for a wide range of coding genes [58, 60] it has also been documented for the non-coding genome [61, 62] such as for imprinted microRNA gene clusters found both on mouse and human chromosomes [59, 63]. In parallel, the preference for the monoallelic expression of microRNA genes could be attributed to differential methylation of regulatory elements controlling gene expression.

Regarding the subnuclear spatiotemporal localization of the microRNA gene loci we studied, our analysis indicated that in both naïve, non-stimulated cells, but also in TCR-activated CD4^+^ T cells or LPS-induced macrophages, microRNA genes localize in the nuclear periphery, irrespectively of their transcriptional activity or differentiation status. The role of the nuclear periphery in gene expression control still remains unclear. Nowadays, the capacity of high-resolution capacity imaging approaches [64–66] in concert with chromosome conformation capture (3C)-based technologies [67], combined with modeling for interpreting chromatin interactions, or tethering loci to specific nuclear compartments [68] provides strong support in deciphering the exact role of the nuclear periphery during development.

Although accumulating evidence supports the notion that gene transcription might be linked with repositioning into the nuclear interior [38–40], several studies have shown that nuclear periphery can also be a transcriptionally “active environment” [35, 42, 69]. Despite the fact that transcription-induced relocalization of protein-coding genes or lamina associated enhancers [41, 69–72] were shown to precede the actual transcription-mediated responses in the murine immune system [41, 43, 44, 73] we have not observed such relocalization of microRNA gene loci before and after activation/differentiation of T cells and macrophages. It still remains unresolved whether transcription actually controls chromatin organization and gene repositioning or *vice versa*. It has been reported that chromatin loops [8] and contacts between regulatory DNA elements and genes in the β-globin locus were unaffected upon transcription inhibition. These reports are in line with our findings in which transcription inhibition did not affect the localization of microRNA genes nor caused a repositioning in T cells.

Perinuclear positioning of microRNA genes was not affected by changes in chromatin conformation during murine development. Our results in bone marrow cells and ESCs support that subnuclear localization of these microRNA gene loci is conserved between embryonic stem cells and terminally differentiated immune cells. In contrary, the protein-coding *Tnfα* gene locus altered its subnuclear localization during development. However, the unique and intriguing chromatin landscape of ESCs has been well documented [74–76], with their nuclear periphery being at the same time a transcriptionally permissive and repressive compartment [77]. Core pluripotency genes expressed in ESCs, such as *Sox2*, *Nanog* and *Oct4* avoid peripheral localization [78], whereas up-regulated genes such as *Ptn*, *Sox6*, and *Nrp1* relocate from the nuclear periphery to the nuclear center during ESC differentiation [12, 79]. These reports are in line with our findings, where *miR-17-92* cluster, which is perinuclearly localized from embryonic stem cells to differentiated myeloid cells, was highly expressed in mESCs [80].

Studies in *S*.*cerevisiae* and *D*.*melanogaster* indicated that active transcription occurs in the NPC vicinity, causing chromatin recruitment to the nuclear periphery [14, 16, 81]. In addition to nuclear transport, NUP153 and NUP93 are considered as gene expression regulators since they control cell type specific gene expression transcriptional programs [82, 83]. Depletion of NUP153 does not affect nuclear transport, though it induces changes in gene transcription [15]. DamID experiments reported that these nucleoporins interact with ciLAD domains and bind to SE in U2OS and IMR-90 cells [83]. These findings are in accordance with our observations regarding the transcriptionally active microRNA genes at the nuclear periphery. In addition, NUP98 also regulates gene expression [84]. It has been reported that in *D*.*melanogaster* NUP98 interacts with transcriptionally active developmental and cell cycle associated genes in the nucleoplasm [13]. This nucleoporin is characterized as a more dynamic molecule that can detach from the nuclear periphery and bind to promoters at a distance from the NPC [85]. This is in accordance with our findings suggesting no significant differences in NUP98 binding between the peripheral microRNA loci and all the protein-coding genes. Therefore, a potential explanation for the perinuclear localization of microRNA gene loci might be their interaction with NPCs.

Our comparative analysis of DamID-derived sequencing data revealed that microRNA genes are preferentially positioned outside constitutively-repressed LAD regions in *M*.*musculus* and this localization pattern is conserved among four different species (*H*.*sapiens*, *M*.*musculus*, *C*.*elegans* and *D*.*melanogaster*). We also documented that both microRNA genes and protein-coding genes display the same preference to avoid cLAD localization. This result is compatible with our immuno-RNA-DNA FISH analysis, showing that microRNA genes are actively transcribed even when colocalized with Lamin-B1. This is in line with previous reports documenting transcriptionally active gene loci in concert with ciLAD-localization [12, 86]. Conclusively, our results are in accordance with reports showing that the frequency of gene colocalization with the nuclear lamina can be locus-specific [87] and that in general microRNA gene colocalization with the nuclear lamina is not associated with attenuated pri-microRNA transcription.

Ultimately, the peripheral positioning of microRNA gene loci might also represent an evolutionary aspect of chromatin organization. cLAD domains are enriched for LINE (Long Interspersed Elements) sequences [36] and the evolution of microRNA genes is speculated to descent from transposable elements integration into the genome [88, 89]. Next-generation sequencing and computational comparative analyses of small RNAs have demonstrated that sequence homology is more frequent for MITEs (Miniature Inverted-repeat Transposable Elements) and DNA transposons and less prevalent for LTR (Long Terminal Repeat) or non-LTR retrotransposons (SINEs, LINEs) or satellite repeats, supporting the notion of microRNA gene derivation from transposable elements [88, 90]. Additionally, it was documented that in human CD4^+^ cells, HIV-1 integrates preferentially in regions of the eukaryotic genome found at the nuclear periphery, in proximity to nuclear pores, avoiding the heterochromatic LAD regions in order to facilitate the transcriptional regulation of the viral genome [91]. These findings provide additional evidence for the evolutionary denouements of peripheral localization and reconfirm the subnuclear positioning of the microRNA genes observed in our study.

In conclusion, our report elucidates the interplay between subnuclear localization of microRNA gene loci and their transcriptional regulation in the murine innate and adaptive immune system, providing valuable additional knowledge of chromatin organization and gene regulation at the nuclear periphery.

## Materials and methods

### Ethics

All procedures were conducted according to Greek national legislations and institutional policies upon ethical committee approval. Mice were maintained at the Institute of Molecular Biology and Biotechnology (IMBB) animal facility following the institutional guidelines based on the Greek ethical committee of animal experimentation, approved by the General Directorate of Veterinary Services, Region of Crete (license number: EL91BIO-02). *Bach1*^*-/-*^ mice were kindly provided by Dr. Kazuhiko Igarashi (Tohoku University). Mice were bedded in sawdust cages with up to three cage mates and exposed to a 12-hour light-dark cycle (dark from 19:00 to 07:00 h). The room temperature where the animals were housed was stable at 23°C and room humidity was 50%.

### Cell culture

Thymocytes were isolated from 4-week old mice, and peripheral CD4^+^ cells were obtained from axillary, popliteal lymph nodes and spleens of 5-6-week-old mice by positive selection with CD4 MicroBeads and MACS separation columns. Differentiation of naive CD4^+^ T cells was carried out by stimulation with plate-bound *α*CD3 and *α*CD28 antibodies. Cells were cultured in CLICK’s medium supplemented with 10% FBS, 100 μg/ml Penicillin/Streptomycin, 0.05 mM β-mercaptoethanol, 2 mM L-Glutamine and 25 mM HEPES for five to six days. For TH1 cell differentiation, culture media were supplemented with 20 units/ml IL-2, 3.5 μg/ml IL-12 and 10 mg/ml IL-4. For TH2 cell differentiation 20–50 units/ml of IL-2, 10 μg/ml IL-4 and 10 mg/ml IFNγ were used. Re-stimulation of terminally differentiated TH1 and TH2 cells was carried out with *α*CD3 plate-bound antibodies for one hour. Cell identity upon opposing conditions of differentiation was further assessed by qRT-PCR analysis for the expression of *Il4* and *Ifnγ* genes. Peritoneal macrophages were elicited from 10-week old mice, 4 days after intraperitoneal treatment with thioglycollate medium, and *in vitro* stimulated at various timepoints with 50 ng/mL LPS (Invivogen). Bone marrow cells were isolated from 10-week old mice femurs and tibia and cultured with 30% custom-made L929 conditioned media for 7 days to achieve a complete differentiation towards the macrophage lineage. Macrophages were cultured in Dulbecco's Modified Eagle's Medium supplemented with 10% FBS, 1% L-Glutamine, 1% penicillin and streptomycin and stimulated at various timepoints with 50 ng/mL LPS (Invivogen). Macrophage cell identity was further assessed with both immunofluorescence and flow cytometry analyses for the expression of CD11b and F4/80 macrophage-specific surface markers. The feeder-independent mouse embryonic stem cell lines JM8.N4 and CGR8 were cultured on 0.1% gelatinized tissue culture plates using the Dulbecco's Modified Eagle's Medium supplemented with 15% fetal bovine serum, 1% L-Glutamine, 1% penicillin and streptomycin, 500 U/ml leukemia inhibitory factor and 100 μM β-mercaptoethanol.

### cDNA synthesis and RT-PCR

For quantitative pri-miRNA expression analysis following *α*-amanitin treatment, whole cell RNA was prepared using the TRI-REAGENT (SIGMA, T9424) following the manufacturer’s instructions. In order to eliminate DNA contamination, RNA samples were treated with DNase I (New England Biolabs, M0303L) for 3 hours at 37°C. cDNA synthesis was performed using 500 ng precipitated RNA and 5pmol of oligo-d(T) primer. For each reverse transcription reaction 200 units M-MuLV Reverse Transcriptase (NEB, M0253S) were used. In parallel with reverse transcriptase reactions, control reactions devoid of the enzyme were prepared in order to verify the absence of DNA contamination in the subsequent quantitative PCR (qPCR) reactions. 10% of the cDNA produced was used for qPCR using the SYBR Green PCR Master mix (Applied Biosystems, Cat.No.4309155) according to the manufacturer’s instructions. Normalization was performed utilizing *Hprt1* mRNA levels. The primer sets used for pri-miRNA quantitation were the following: *miR-142*.F 5’-GAAGAATCCCCGTGGACAGA-3’, *miR-142*.R 5’-CCCAAGTATCAGGGGTCAGG-3’, *miR-146a*.F 5’-GCCAGCCCTGTAAAAACACA-3’, *miR-146a*.R 5’-TCTTCGCTGGGATTATGGGG-3’, *miR-155*.F 5’-ACCCTGCTGGATGAACGTAG-3’, *miR-155*.R 5’-CATGTGGGCTTGAAGTTGAG-3’, *Hprt1*.F 5’-GTCCCAGCGTCGTGATTAGC-3’, *Hprt1*.R 5’-TTCCAAATCCTCGGCATAATG-3’

### 3D fluorescence *in situ* hybridization (RNA-DNA FISH)

To remove the cytoplam, cells were treated for either 2.5 minutes (thymocytes and CD4) or 3 minutes (TH1, TH2 and BMDMs) or 4 minutes (TEPMs) with cytoskeletal buffer (CSK) containing 100mM NaCl, 300mM sucrose, 3mM MgCl2, 10mM PIPES, 0.5% Triton X-100, 1mM EGTA, and 2mM vanadyl-ribonucleoside complex. Cells were then fixed with 4% PFA/1X PBS for 10 minutes, washed three times with 70% ethanol. For each hybridization 100ng from each probe (DNA and RNA probes), 1μg mouse COT-1 DNA and 20μg yeast transfer RNA were lyophilized for 15 minutes at 45°C, resuspended in 5μl de-ionized formamide and incubated at 37°C for one hour. Probes were further denatured at 95°C for 10 minutes and then thoroughly mixed with 5μl of 2X hybridization buffer (4X SSC, 20% Dextran sulfate, 2mg/ml acetylated BSA, 50mM Sodium Phosphate). Cells were then dehydrated through an ethanol series and hybridization was done overnight at 37°C after either 2.5 minutes (thymocytes, CD4) or 4 minutes (TH1, TH2, TEMPs and BMDMs) denauration at 73°C. After the post hybridization washes, a tyramide signal ampilificaion (TSA) of primary microRNA transcripts was performed. Tyramide signal amplification reaction was performed using TSA biotin system kit (Perkin Elmer). More specifically, cells were blocked for 30 minutes with TNB blocking buffer (100mM Tris-HCl pH 7.5, 150mM NaCl, 0.5% blocking reagent provided with the kit) and then incubated with streptavidin (SA), conjugated with horseradish peroxidase (HRP) in a dilution of 1/200 in TNB for additional 30 minutes at RT. The hybridized cells were washed twice with TNT buffer (100mM Tris-HCl pH 7.5, 150mM NaCl, 0.05% Tween 20) for 3 minutes at RT. The cells were then incubated for 10 minutes at RT with biotinylated tyramide in a dilution of 1/50 in amplification diluent and rinsed twice with TNT buffer for 3 minutes. For the visualization of the amplified RNA signal, cells were incubated with fluorophore-conjugated SA-488 for 30 minutes at RT, washed twice with TNT buffer and once with 1X PBS for 3 minutes each and mounted with ProLong Gold antifade reagent containing 4′,6-diamidino-2-phenylindole (DAPI).

### 3D fluorescence *in situ* hybridization (DNA FISH)

Cells were fixed with 4% PFA/1X PBS for 10 minutes, permeabilized with 0.5% Triton X-100 for 5 minutes, rinsed with 1X PBS and incubated in 20% glycerol/1X PBS for 30 minutes. After three freeze-thaw cycles in liquid nitrogen, cells were incubated in 0.1N HCl for 5 minutes, rinsed with 2X SSC and dehydrated through an ethanol series. DNA probe (0.1 μg) was denatured for 5 min at 95°C and applied to coverslips. Hybridization of fixed cells was performed overnight at 37°C after 5 minutes denaturation at 73°C. Cells were rinsed three times with 2X SSC, once with 1X PBS and mounted in ProLong Gold containing DAPI (Invitrogen).

### RNA FISH probes

cDNA probes were constructed to detect the pri-microRNA transcript. The procedure entailed the creation of 1kb probes cloned into a TOPO® TA vector. BAC DNA containing each microRNA gene was used as template for the amplification of 1kb PCR fragments encompassing the manure microRNA genomic region. The primer sets used for PCR amplification are listed below. The ~1kb PCR products were subsequently purified and cloned into a TOPO® TA vector. The isolated cloned plasmids were used for the preparation of the cDNA FISH probes. To confirm the cDNA probe specificity, we conducted RNA FISH on RNAse A treatment cells for 45 minutes. Furthermore, the empty TOPO® TA vector was also used for the preparation of biotinylated probes and used as a negative control. Primers used for the amplification of the 1Kb pri-microRNA fragments: *miR-181a1b1*.F: 5’-CATGCGTCCTTGCAGTTCTTT-3’, *miR-181a1b1*.R: 5’-GGATAACGGGGCAGGAAGTA-3’, *miR-181a2b2*.F: 5’-GTGAGAGACCCAACAGCAG-3’, *miR-181a2b2*.R: 5’-CCTGGAGCAGTACTTCCGTA-3’, *miR-181c*.F: 5’-GGTCGATGGTTTGTCTGAGC-3’, *miR181c*.R: 5’-GCAGGAGTTTCACACAAGCA-3’, *miR-142*.F: 5’-GCCATTTCTGCCAACACACT-3’, *miR-142*.R: 5’-CCCCAGGCTGTGTCTTAGTC-3’, *miR-146a*.F: 5’-AGCACTGTCAACCTGACACA-3’, *miR-146a*.R: 5’-GGACCAGCAGTCCTCTTGAT-3’, *miR-17/92*.F: 5’-ACTTCTGGCTATTGGCTCC-3’, *miR-17/92*.R: 5’-AACTTCACCTAAGCCCCCAC-3’, *miR-155*.F: 5’-CGGTTTGTGAGTCCCCAAAG-3’, *miR-155*.R: 5’-ATGTCAGTCGAGAATGGCCG-3’, *miR-let7e*.F: 5’-CACCCTCCCTACTTCTGGTC-3’, *miR-let7e*.R: 5’-AAAGGAACCAGGAGATGCCT-3’.

### *α*-Amanitin treatment

Thymocytes, CD4^+^ and TH2 cells were treated with Click’s culture media supplemented with 10% FBS, 100μg/ml Penicillin/Streptomycin, 0.05mM β-mercaptoethanol, 2mM L-Glutamine, 25mM HEPES, 20–50 U/ml IL-2 and 50μg/ml *α*-amanitin for 6 hours at 37°C. Cells treated under the same conditions without *α*-amanitin were used as a control. CGR8 embryonic stem cells were cultured as previously described and treated with *α*-amanitin for 6h.

### Immuno-DNA-FISH

Combined detection of LMNB1 and microRNA genes was carried out and the procedure followed was a combination of the DNA FISH and Immunofluorescence (IF) analysis. After DNA FISH hybridization, the cells were blocked with IF blocking buffer (1% BSA/1X PBS) for 30 minutes, incubated with the primary antibody (LaminB1 or Lamin A/C 1:200) for 1 hour, with the secondary antibody (Invitrogen 1:500) for 45 minutes, rinsed three times with 1X PBS and mounted as above.

### Immuno-RNA-FISH

For the IF/RNA-DNA FISH experiments the steps followed included RNA/DNA FISH (as previously described) prior to IF labeling as described in the IF/DNA FISH section.

### Confocal microscopy and quantitative 3D image analysis

Images were acquired using inverted laser scanning confocal microscope with spectral detection (Leica TCS SP8 microscope unit). Maximum projections and deconvolution of the images were performed with Volocity software (Perkin Elmer) by two different investigators. Images of ~25–40 optical sections were captured with a 63x objective (step of 250nm) and acquired at different λs. A 405nm laser was used to excite and detect DAPI staining, whilst 488-nm, 561-nm and 633-nm lasers were used to excite and detect Alexa Fluor 488, Alexa Fluor 561 and Alexa Fluor 647 respectively in separate sequence to avoid bleed-through in different channels. Maximum projections and deconvolution of the images were performed with the Volocity software (Perkin Elmer) by two independent investigators. Intranuclear distances between microRNA gene alleles and the edges of the cell nucleus, were determined from the center of the gene allele to the edge of the cell border, for all DNA- RNA-FISH and IF experiments. The absolute distances were normalized by the nuclear radius for each scored cell. In order to analyze and visualize the normalized distances, we clustered them in 10 concentric circles of equal area (0–0.1, 0.1–0.2, 0.2–0.3, 0.3–0.4, 0.4–0.5, 0.5–0.6, 0.6–0.7, 0.7–0.8, 0.8–0.9, 0.9–1) as presented in S2A Fig where 0 and 1 were determined as the nuclear periphery and the nuclear center respectively. The aforementioned measurements have only been performed for images depicting nuclei with two signals (alleles) and having an intact 3D architecture. For all cell types under investigation, at least 120 nuclei were analyzed per biological replicate. Furthermore, the positions of microRNA gene alleles relative to LMNB1 were also determined through the analysis of 3D-preserved nuclei using the Volocity software. microRNA gene allele (red) and LMNB1 protein (green) colocalization was determined either by juxtaposition and partial overlap of red and green pixels or complete overlap between red and green pixels.

### Statistical analysis

Statistical analysis for the randomness of allelic distance distribution was performed using the non-parametric two-tailed Kolmogorov-Smirnov test. The reported *p* and *D* values were calculated with the XL-STAT and GraphPad Prism 7 software package. *The p*-value for statistically significant differences in allele distribution comparisons was set to p< 0.0001. Welch’s T-test was used to test significant differences between microRNA within and outside of cLADs. Pearson r was used to study the linear correlation between microRNA within or outside cLADs and gene density. For data presentation, graphs were designed using GraphPad Prism 7 and Sigma Plot Software.

### Bioinformatic analysis of microRNA cLAD/ciLAD co-localization characterization and distance distribution assessment

Datasets used for the analysis of microRNA cLAD/ciLAD co-localization: mouse pre-miRNA gene coordinates were downloaded from miRBase (Release 21) for four different species (mouse: *Mus musculus*, human: *Homo sapiens*, fly: *Drosophila melanogaster*, worm: *Caenorhabditis elegans*). LAD coordinates for the same species were obtained from published DamID experiments in human [54], mouse [12], fruit fly [55] and worm [56] respectively. For the mouse genome we obtained constitutive LAD domains through comparison of four different tissues [12]. For the same genome and from the same experiments we obtained the constitutive inter-LAD (ciLAD) domains, termed as the regions that constitute domains intervening defined LADs in the aforementioned tissues studied. Coordinates for both microRNA and cLAD/ciLAD were converted to hg19 (human), mm9 (mouse), dm3 (fly) and ce10 (worm). Protein coding gene coordinates were obtained from the UCSC Genome Browser under the RefSeq catalogues of Genes and Gene Prediction Tracks. For human and mouse genomes we made use of EBI's Expression Atlas to define constitutive protein coding genes. Human constitutive genes were called the ones which were designated as highly expressed in at least 80% of the 32 studied tissues, while tissue-specific ones were called on the basis of the corresponding percentage being less than 10%. For the mouse genome as only 6 tissues were available we only called constitutive genes the ones belonging to the top 10% of expressing genes in all 6 tissues. Analysis of microRNA overlap with cLAD and ciLAD: overlap enrichment analysis was conducted as follows. The total overlap percentage between microRNA gene loci and cLAD/ciLAD was calculated through the intersection of the two coordinate files, using BEDTools [92] intersect function. The enrichment of the observed overlap was calculated as the ratio over an expected value obtained through a simple calculation of intersection, based on the genome coverage percentage of each of the two coordinates. Following this analysis, enrichment values greater than 1 correspond to a greater overlap than the one expected by chance, indicative of co-localization preference. Values below 1 are indicative of an avoidance of co-localization preference. The significance of the enrichment values was calculated in all cases on the basis of a permutation test as previously described [93]. The microRNA gene coordinates were shuffled in the genome in random positions, keeping the distribution of sizes and number of elements unchanged and the observed overlap was calculated. This process was repeated for 1000 such permutations and p-values were set as the ratio of times an overlap as big (or as small) as the initial one was found in the total number of trials (N = 1000). In cases where not even a single random permutation yielded such a high (or low) overlap value, p-value was set to be <1/N, in our case <0.001. Analysis of mouse microRNA distance distribution from cLAD/ciLAD: having observed a clear opposite tendency for co-localization of mouse microRNA genes in cLADs and ciLADs we went on to check whether this tendency was also quantitative in the sense of distance from cLAD/ciLAD boundaries, that is whether microRNAs exhibited preferences towards or away from the regions of transition between the two domain categories. To answer this question for each mouse microRNA gene locus, we calculated the distance to the closest cLAD/ciLAD domain. For microRNA gene loci falling outside the corresponding domains we retained only those that lied within one domain size from the closest boundary (for instance, if a cLAD was 100 kb and a microRNA gene locus was lying >100 kb upstream or downstream of its closest boundary, the microRNA gene was discarded from the analysis). We then scaled the obtained distances by dividing over the cLAD/ciLAD size in order to accommodate the variability in domain sizes. In this way, a microRNA gene locus could either: a) lie within the domain and thus be no farther than half the domain size from its closest boundary, or b) lie outside of the domain and thus, by definition, not farther than one full domain length. As there is no reason to assume a directional effect that distinguishes between the domain boundaries or upstream/downstream localization, we took all distances within the domains as positive and all distances from outside the domain as negative. In this way the scaled microRNA-cLAD/ciLAD distances were defined in a range of [-1, 0.5], with -1 being the farthest possible element outside the domain, 0 being the boundary and 0.5 being the farthest possible towards the center of the domain.

### Analysis of microRNA and mRNA loci overlaps with nucleoporins

An overlap analysis was performed in a way similar to the one described for the cLAD/ciLAD regions. This time the compared coordinates were the ones of protein-coding and microRNA gene loci against the nucleoporin peaks as provided by publicly available datasets.

### RNA/GRO-seq analysis

Depending on the dataset used in each analysis, either normalized gene counts (RPKM) or continuous signal values (in the case of GRO-seq) were implemented. In the case of RNA-seq RPKM values were assigned to each gene that was included in the public dataset. For GRO-seq, provided as continuous scores in the form of bigWig files, we calculated a mean aggregate score along the region that corresponded to each locus’ coordinates as described above.

### GEO accession numbers of datasets used for comparative-analyses

LaminB1 DamID-seq Human: GSE8854, LaminB1 DamID-seq mouse: GSE17051, LaminB1 DamID-seq fruit fly: GSE5089, LEM-2 ChIP-seq worm: GSE25933, RNA-seq mouse ESCs: GSM2095053, RNA-seq mouse Thymocytes: GSM2095060, RNA-seq mouse CD4 cells: GSM1120730, RNA-seq mouse TH1 cells: GSM1120731, https://www.ncbi.nlm.nih.gov/geo/query/acc.cgi?acc=GSM1120731RNA-seq mouse TH2 cells: GSM1120732, RNA-seq mouse BMDMs: GSM940701, GRO-seq mouse ESCs & MEFs: GSE27037, NUP 153 DamID-seq: GSE64008, UP-98 ChIP-seq: GSE48996, NUP-93 DamID-seq: GSE87831.

## Supporting information

S1 Fig Relative expression levels of genes within the BAC clones used for DNA/ RNA-DNA FISH experiments of microRNA genes. (A) Bar graphs depicting the relative mRNA levels of both coding and non-coding genes flanking each of the eight microRNA genes under study in each BAC clone. RPKM (Reads Per Kilobase per Million) values calculated from publicly available RNA-seq datasets for the six cell types indicated. (B) Nascent transcription of the genes as in (A) as deduced from aggregate score analysis of publicly available GRO-seq data in CD4^+^ T-cells and mouse embryonic fibroblasts (MEFs).
(TIF)

S2 Fig Statistical analysis for the perinuclear distribution of microRNA gene loci. (A) Compartmentalization of measured nuclei based on normalized distances (NDs). Distance between microRNA gene alleles and the edge of the cell nucleus as deduced by DAPI staining were normalized to the nuclear radius yielding 10 concentric shells. ND = 1 defines the center of the nucleus, whereas ND = 0 is indicative of the nuclear periphery. (B) Cumulative frequency graph of calculated allele ND values, following a reversed distribution pattern compared to the *Tnfα* control in thymocytes, CD4^+^, TH1, TH2 cells and TEPMs (naive and LPS-stimulated). Kolmogorov Smirnov (KS) non-parametric analysis, showing that the allelic distributions of microRNA gene allele ND values differ from *Tnfα* allele NDs (p>0.05). *p*- and D-values characterizing each distribution are depicted in the table. The relative cumulative frequency values of distributions are depicted on the y-axis, whereas their corresponding ND values on the x-axis. KS-test *p*-values are separately depicted for each distribution comparison. (C) Single z-stack confocal images of DNA FISH analysis indicating the perinuclear localization of the microRNA genes tested compared to the internal spatial distribution of the *Tnfα* control locus. Scale bar 2μm. Box plots displaying the quantitative analysis of the intranuclear 3D distance between each allele and the nuclear periphery in naive and LPS-stimulated BMDMs. The reported *p*-values were calculated using the XL-STAT software package. Allele distribution differences were calculated with the Kolmogorov-Smirnov test analysis (p<0.0001). (D) Bar graphs representing the frequency of cells bearing at least one allele associated with nuclear lamina in naive (0h) and LPS (2h, 6h) stimulated BMDMs. The total number of nuclei deployed in these measurements were: *miR-146a*: n = 101 (0h), n = 263 (2h), n = 344 (6h), *miR-155*: n = 112 (0h), n = 215 (2h), n = 122 (6h) and *miR-let7e*: n = 33 (0h), n = 86 (2h), n = 48 (6h).
(TIF)

S3 Fig Perinuclear microRNA gene loci localization irrespective of their transcription activity. Distribution of non-expressing and expressing gene allele ND values. The normalized distances (y-axis) indicated in each box plot for each microRNA gene are characterized by their median. 95% of the ND values are included within the whiskers of each box plot, whereas single allele outliers (remaining 5% of total NDs) are indicated in the vicinity outside the whiskers. (A) T cells: Thymocytes, CD4^+^, TH1 and TH2 cells. Total number of alleles analyzed for each dataset were n = 623, n = 807, n = 497, n = 472 for thymocytes, CD4^+^, TH1 and TH2 cells, respectively. (B) TEPMs and BMDMs before and after LPS stimulation. The total allele number contained in each dataset were n = 1154 for TEPMS and n = 2652 for BMDMs. (C) Relative mRNA expression corrected to *Hprt1* mRNA levels of *pri-miRNA-146α*, *-142*, *-155* with/or without *α*-amanitin treatment in thymocytes, CD4^+^ and TH2 cells. (D) Allelic expression profile of the indicated microRNA genes as deduced by RNA-DNA FISH analysis before and after transcriptional inhibition of cells with *α*-amanitin. (E) KS-test results related to gene alleles ND distribution presented in Fig 3D. (F) KS-test results related to gene alleles ND distribution presented in Fig 3E. (G) Comparison of microRNA and protein coding mRNA gene loci coordinates against peaks from human NUP98. Enrichment was calculated as observed over expected ratios of overlaps between the compared genomic regions. P-value was calculated on the basis of 1000 random permutations of the peaks’ regions. A value of <0.001 indicates that not one out of 1000 permutations had a value as high as the one observed.
(TIF)

S4 Fig Perinuclear distribution of microRNA gene loci in non-differentiated cells. (A) Perinuclear distribution of microRNA genes and *Tnfα*, as a control locus, in CGR8 embryonic stem cells. Single z-stack DNA FISH images portraying the peripheral localization of microRNA gene loci (red). KS non-parametric analysis, showing that the allelic distributions of microRNA gene allele ND values compared to *Tnfα* are alike (p>0.05). P- and D-values characterizing the compared distributions are depicted. The relative cumulative frequency values of compared distributions are depicted on the y-axis, whereas their corresponding ND values on the x-axis. KS-test p-values are separately depicted for each distribution comparison. The analysis was performed in n = 208 total alleles for *miR-181a1b1*, n = 206 for *miR-181a2b2*, n = 240 for *miR-181c*, n = 214 for *miR-142*, n = 188 for *miR-146a*, n = 130 for *miR-17-92*, n = 158 for *miR-155*, n = 214 for *miR-let7e* and n = 160 alleles for *Tnfα* respectively. (B) KS-test indicating the statistically significant (p<0.001) differences of microRNA gene allele distributions compared to *Tnfα* in bone marrow cells. A total of 2044 alleles were counted. (C) KS-test results related to gene allele ND distribution presented in Fig 5A for JM8.N4 ESCs. A total of 1968 alleles were measured. (D) Bar graph representing the gene density for each chromosome of *M*.*musculus*, *H*.*sapiens*, *D*. *melanogaster* and *C*.*elegans*.
(TIF)

S1 Table Allelic expression profile of genes localized on BAC clones utilized for DNA FISH of microRNA gene loci. Monoallelic expression (blue), biallelic expression (red), undetermined (black), according to "dbMAE: the database of autosomal monoallelic expression". Assembly used for coordinates: GRCm38.p3 (C57BL/6J) [94].
(PDF)

S1 Video 3D-DNA FISH experiment in CD4^+^ cells. Visualization of a representative 3D-DNA FISH experiment in CD4^+^ cells demonstrating *miR-155* gene locus that is preferentially located at the nuclear periphery. *miR-155* gene locus (red) was labeled with Alexa-594 and CD4^+^ cell DNA counterstained with DAPI (blue). The video was created based on raw confocal microscopy data with the Volocity software (Perkin Elmer).
(MOV)

S2 Video 3D-IF/DNA FISH in CD4^+^ cells. Visualization of a representative 3D-IF/DNA FISH in CD4^+^ cells demonstrating the colocalization of *miR-155* gene locus with the nuclear lamina. As shown here, for most CD4^+^ cells, *miR-155* alleles are completely embedded in Lamin-B1-stained regions. *miR-155* gene locus (red) was labelled with Alexa-594, Lamin-B1 was labeled with Alexa-488 and CD4^+^ cell DNA counterstained with DAPI (blue). The video was created based on raw confocal microscopy data with the Volocity software (Perkin Elmer).
(MOV)
