# Supplementary figures and images for: Developmental conservation of microRNA gene localization at the nuclear periphery
Source: PLoS One. 2019 Nov 4;14(11):e0223759. doi: 10.1371/journal.pone.0223759 (PMC6827902; doi:10.1371/journal.pone.0223759)

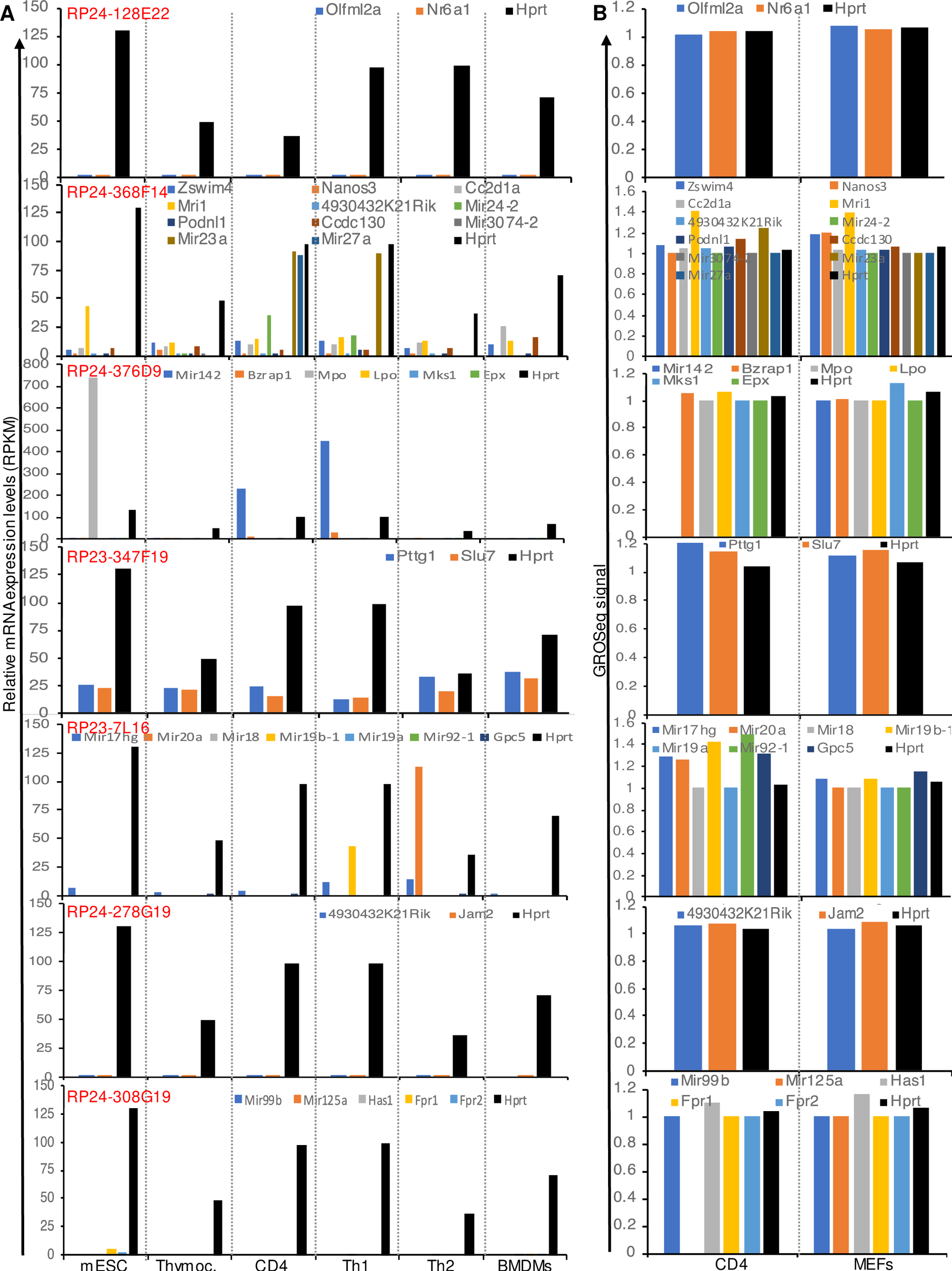

Supplement: S1 Fig — (A) Bar graphs depicting the relative mRNA levels of both coding and non-coding genes flanking each of the eight microRNA genes under study in each BAC clone. RPKM (Reads Per Kilobase per Million) values calculated from publicly available RNA-seq datasets for the six cell types indicated. (B) Nascent transcription of the genes as in (A) as deduced from aggregate score analysis of publicly available GRO-seq data in CD4+ T-cells and mouse embryonic fibroblasts (MEFs). (TIF) [file pone.0223759.s001.tif]

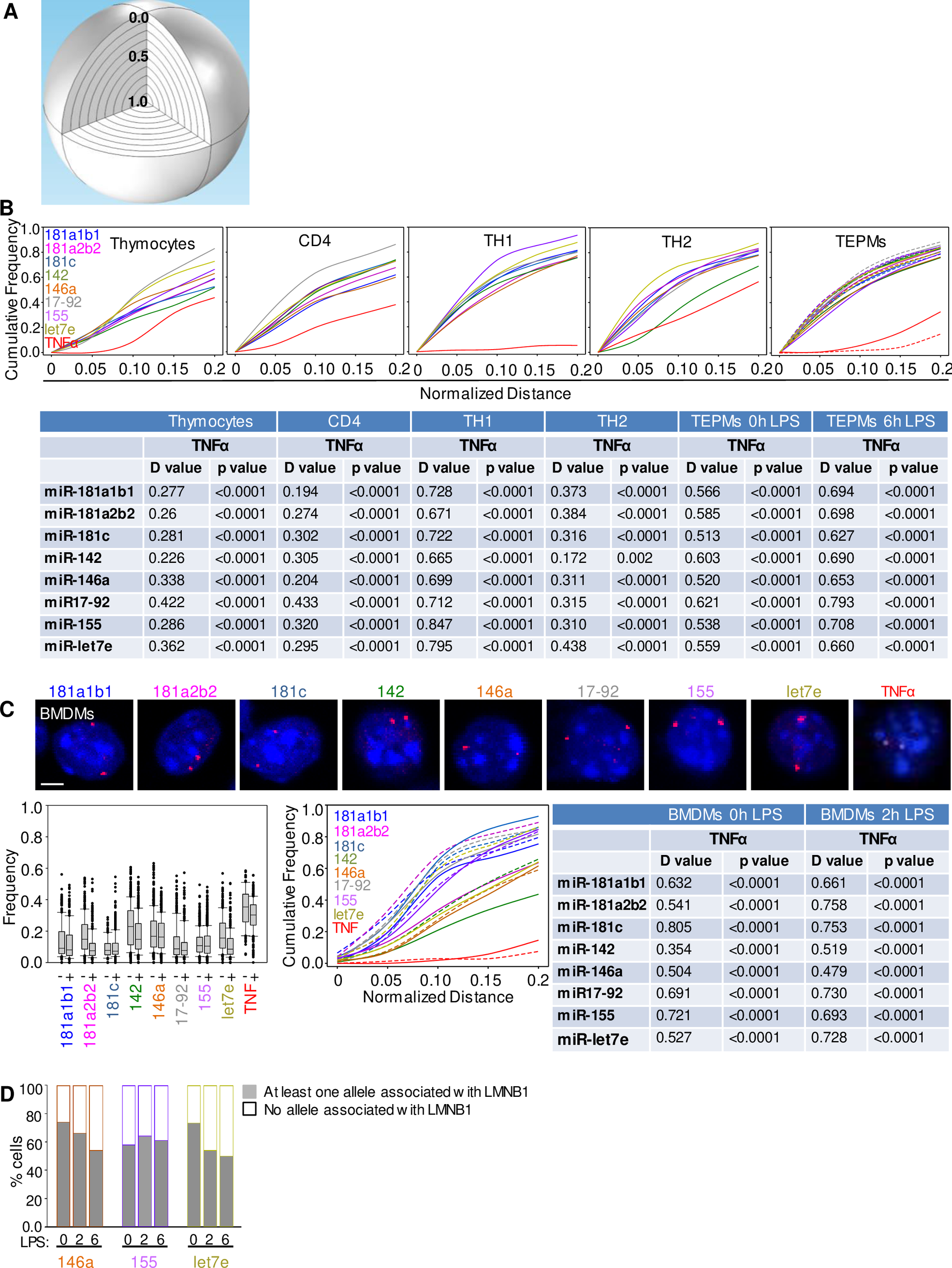

Supplement: S2 Fig — (A) Compartmentalization of measured nuclei based on normalized distances (NDs). Distance between microRNA gene alleles and the edge of the cell nucleus as deduced by DAPI staining were normalized to the nuclear radius yielding 10 concentric shells. ND = 1 defines the center of the nucleus, whereas ND = 0 is indicative of the nuclear periphery. (B) Cumulative frequency graph of calculated allele ND values, following a reversed distribution pattern compared to the Tnfα control in thymocytes, CD4+, TH1, TH2 cells and TEPMs (naive and LPS-stimulated). Kolmogorov Smirnov (KS) non-parametric analysis, showing that the allelic distributions of microRNA gene allele ND values differ from Tnfα allele NDs (p>0.05). p- and D-values characterizing each distribution are depicted in the table. The relative cumulative frequency values of distributions are depicted on the y-axis, whereas their corresponding ND values on the x-axis. KS-test p-values are separately depicted for each distribution comparison. (C) Single z-stack confocal images of DNA FISH analysis indicating the perinuclear localization of the microRNA genes tested compared to the internal spatial distribution of the Tnfα control locus. Scale bar 2μm. Box plots displaying the quantitative analysis of the intranuclear 3D distance between each allele and the nuclear periphery in naive and LPS-stimulated BMDMs. The reported p-values were calculated using the XL-STAT software package. Allele distribution differences were calculated with the Kolmogorov-Smirnov test analysis (p<0.0001). (D) Bar graphs representing the frequency of cells bearing at least one allele associated with nuclear lamina in naive (0h) and LPS (2h, 6h) stimulated BMDMs. The total number of nuclei deployed in these measurements were: miR-146a: n = 101 (0h), n = 263 (2h), n = 344 (6h), miR-155: n = 112 (0h), n = 215 (2h), n = 122 (6h) and miR-let7e: n = 33 (0h), n = 86 (2h), n = 48 (6h). (TIF) [file pone.0223759.s002.tif]

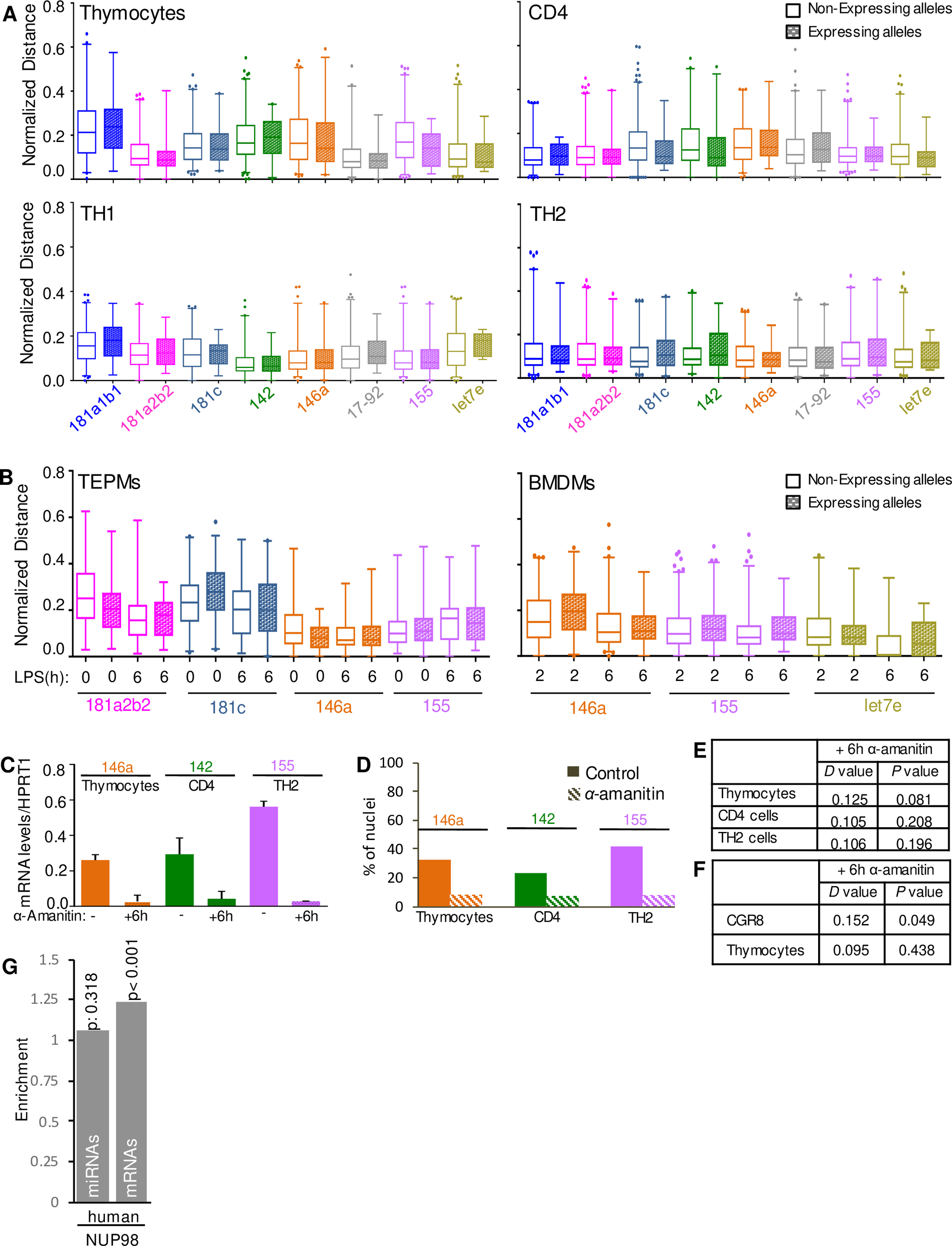

Supplement: S3 Fig — Distribution of non-expressing and expressing gene allele ND values. The normalized distances (y-axis) indicated in each box plot for each microRNA gene are characterized by their median. 95% of the ND values are included within the whiskers of each box plot, whereas single allele outliers (remaining 5% of total NDs) are indicated in the vicinity outside the whiskers. (A) T cells: Thymocytes, CD4+, TH1 and TH2 cells. Total number of alleles analyzed for each dataset were n = 623, n = 807, n = 497, n = 472 for thymocytes, CD4+, TH1 and TH2 cells, respectively. (B) TEPMs and BMDMs before and after LPS stimulation. The total allele number contained in each dataset were n = 1154 for TEPMS and n = 2652 for BMDMs. (C) Relative mRNA expression corrected to Hprt1 mRNA levels of pri-miRNA-146α, -142, -155 with/or without α-amanitin treatment in thymocytes, CD4+ and TH2 cells. (D) Allelic expression profile of the indicated microRNA genes as deduced by RNA-DNA FISH analysis before and after transcriptional inhibition of cells with α-amanitin. (E) KS-test results related to gene alleles ND distribution presented in Fig 3D. (F) KS-test results related to gene alleles ND distribution presented in Fig 3E. (G) Comparison of microRNA and protein coding mRNA gene loci coordinates against peaks from human NUP98. Enrichment was calculated as observed over expected ratios of overlaps between the compared genomic regions. P-value was calculated on the basis of 1000 random permutations of the peaks’ regions. A value of <0.001 indicates that not one out of 1000 permutations had a value as high as the one observed. (TIF) [file pone.0223759.s003.tif]

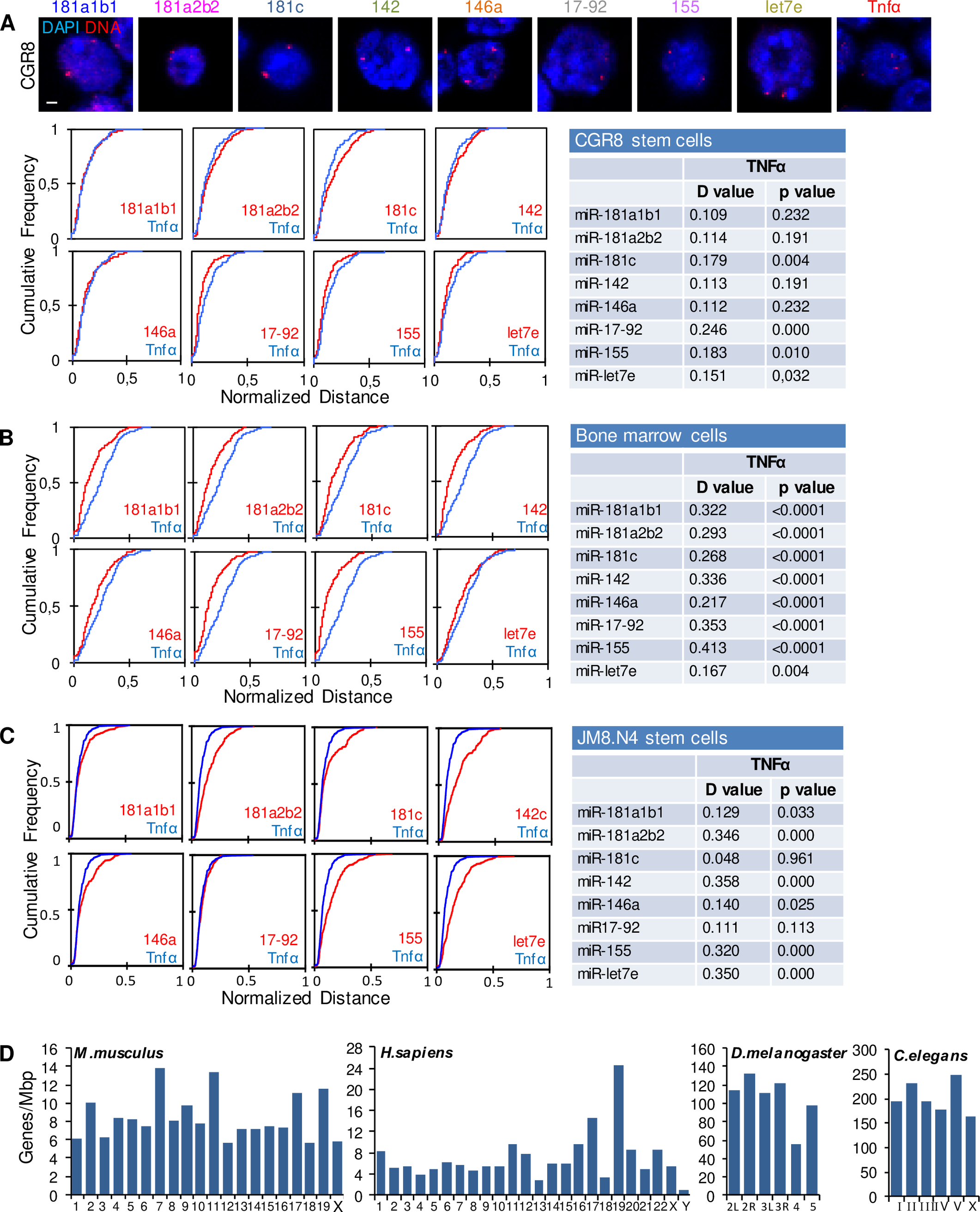

Supplement: S4 Fig — (A) Perinuclear distribution of microRNA genes and Tnfα, as a control locus, in CGR8 embryonic stem cells. Single z-stack DNA FISH images portraying the peripheral localization of microRNA gene loci (red). KS non-parametric analysis, showing that the allelic distributions of microRNA gene allele ND values compared to Tnfα are alike (p>0.05). P- and D-values characterizing the compared distributions are depicted. The relative cumulative frequency values of compared distributions are depicted on the y-axis, whereas their corresponding ND values on the x-axis. KS-test p-values are separately depicted for each distribution comparison. The analysis was performed in n = 208 total alleles for miR-181a1b1, n = 206 for miR-181a2b2, n = 240 for miR-181c, n = 214 for miR-142, n = 188 for miR-146a, n = 130 for miR-17-92, n = 158 for miR-155, n = 214 for miR-let7e and n = 160 alleles for Tnfα respectively. (B) KS-test indicating the statistically significant (p<0.001) differences of microRNA gene allele distributions compared to Tnfα in bone marrow cells. A total of 2044 alleles were counted. (C) KS-test results related to gene allele ND distribution presented in Fig 5A for JM8.N4 ESCs. A total of 1968 alleles were measured. (D) Bar graph representing the gene density for each chromosome of M.musculus, H.sapiens, D. melanogaster and C.elegans. (TIF) [file pone.0223759.s004.tif]
