# Supplementary material for: Developmental conservation of microRNA gene localization at the nuclear periphery
Source: PLoS One. 2019 Nov 4;14(11):e0223759. doi: 10.1371/journal.pone.0223759 (PMC6827902; doi:10.1371/journal.pone.0223759)
Supplement: S1 Table — Monoallelic expression (blue), biallelic expression (red), undetermined (black), according to "dbMAE: the database of autosomal monoallelic expression". Assembly used for coordinates: GRCm38.p3 (C57BL/6J) [94]. (PDF) [file pone.0223759.s005.pdf]

miR	Chr	BAC clone	BAC clone Coordinates	miR coordinates	Other genes
**miR-181a1/b1**	1	RP24-71D3	137,857,577 - 138,069,947	a1:137,966,455-137,966,541 b1:137,966,639-137,966,718	
**miR-181a1/b1**	2	RP24-128E22	38,773,611 - 38,951,434	a2: 38,852,735-38,852,810 b2: 38,853,830-38,853,918	Nr6a1, Olfml2a,
**miR-181c**	8	RP24-368F14	84,109,833 - 84,296,280	84,178,873-84,178,961	Podnl1, Cc2d1a, 4930432K21Rik, Nanos3, mir181-d, Zswim4, Ccdc130, Mri1, Mir24-2, Mir27a, Mir3074-2, Mir23a
**miR-142**	11	RP24-376D9	87,718,667 - 87,882,764	87,756,864-87,756,927	mir142, Supt4a, Bzrap1, Mir142b, Mpo, Lpo, Mks1, Epx,
**miR-146a**	11	RP23-347F19	43,271,828 - 43,471,844	43,374,397 - 43,374,461	Pttg1, Slu7,
**miR-17-92**	14	RP23-7L16	114,948,625 - 115,140,014	115,043,671-115,043,754	Mir17hg, Mir20a, Mir18, Mir19b-1, Mir19a, Mir92-1, Gpc5
**miR-155**	16	RP24-278G19	84,640,010 - 83,813,137	84,714,140-84,714,204	4930529L06Rik, Mir155hg, Jam2,
**miR-let7e**	17	RP24-308G19	17,766,734 - 17,911,549	17,830,352 - 17,830,444	Spaca6, Mir99b, Mir125a, Gm36907, Has1, Fpr1, Fpr2,
